# Mutations in the *Plasmodium falciparum* chloroquine resistance transporter, PfCRT, enlarge the parasite’s food vacuole and alter drug sensitivities

**DOI:** 10.1038/srep14552

**Published:** 2015-09-30

**Authors:** Serena Pulcini, Henry M. Staines, Andrew H. Lee, Sarah H. Shafik, Guillaume Bouyer, Catherine M. Moore, Daniel A. Daley, Matthew J. Hoke, Lindsey M. Altenhofen, Heather J. Painter, Jianbing Mu, David J. P. Ferguson, Manuel Llinás, Rowena E. Martin, David A. Fidock, Roland A. Cooper, Sanjeev Krishna

**Affiliations:** 1 Institute for Infection and Immunity, St. George’s, University of London, London SW17 0RE, UK; 2 Department of Microbiology and Immunology, Columbia University Medical Center, New York, NY 10032, USA; 3 Research School of Biology, Australian National University, Canberra, ACT 2601, Australia; 4 Sorbonne Universités, UPMC Univ. Paris 06, UMR 8227, Integrative Biology of Marine Models, Comparative Physiology of Erythrocytes, Station Biologique de Roscoff, Roscoff, France; 5 CNRS, UMR 8227, Integrative Biology of Marine Models, Comparative Physiology of Erythrocytes, Station Biologique de Roscoff, Roscoff, France; 6 Department of Biological Sciences, Old Dominion University, Norfolk, VA 23529, USA; 7 Department of Biochemistry and Molecular Biology and Center for Malaria Research, Pennsylvania State University, State College, Pennsylvania 16802, USA; 8 Laboratory of Malaria and Vector Research, National Institute of Allergy and Infectious Diseases, National Institutes of Health, Rockville MD 20852, USA; 9 Nuffield Department of Clinical Laboratory Sciences, University of Oxford, John Radcliffe Hospital, Oxford OX3 9DU, UK; 10 Division of Infectious Diseases, Department of Medicine, Columbia University Medical Center, New York, NY 10032, USA; 11 Department of Natural Sciences and Mathematics, Dominican University of California, San Rafael, CA 94901, USA

## Abstract

Mutations in the *Plasmodium falciparum* chloroquine resistance transporter, PfCRT, are the major determinant of chloroquine resistance in this lethal human malaria parasite. Here, we describe *P. falciparum* lines subjected to selection by amantadine or blasticidin that carry PfCRT mutations (C101F or L272F), causing the development of enlarged food vacuoles. These parasites also have increased sensitivity to chloroquine and some other quinoline antimalarials, but exhibit no or minimal change in sensitivity to artemisinins, when compared with parental strains. A transgenic parasite line expressing the L272F variant of PfCRT confirmed this increased chloroquine sensitivity and enlarged food vacuole phenotype. Furthermore, the introduction of the C101F or L272F mutation into a chloroquine-resistant variant of PfCRT reduced the ability of this protein to transport chloroquine by approximately 93 and 82%, respectively, when expressed in Xenopus oocytes. These data provide, at least in part, a mechanistic explanation for the increased sensitivity of the mutant parasite lines to chloroquine. Taken together, these findings provide new insights into PfCRT function and PfCRT-mediated drug resistance, as well as the food vacuole, which is an important target of many antimalarial drugs.

Chloroquine (CQ) rapidly became one of the most useful antimalarial drugs for first-line therapy soon after the Second World War. Resistance to CQ was first reported in the late 1950s in *Plasmodium falciparum*. It then spread globally and forced the development of alternative regimes, culminating in the more expensive artemisinin-based combination therapies (ACTs) used today. The locus containing the *P. falciparum* chloroquine resistance transporter gene (*pfcrt*) was initially mapped by classical genetic studies as being crucial to the development of CQ resistance, with this gene subsequently being identified and its role confirmed using reverse genetic approaches^1^,^2^,^3^. CQ resistance is now emerging in *P. vivax*, for which it remains the first-line treatment^4^.

CQ is a diprotic weak base that accumulates in the parasite’s acidic food vacuole (FV) by diffusion and subsequent trapping by protonation. CQ interferes with the detoxification of heme in the FV, which leads to parasite death^5^. Predicted to have 10 transmembrane domains (TMDs), PfCRT is located in the FV membrane^1^,^6^ and, when mutated, increases export of CQ from the FV and its target of heme polymerisation^7^. Single nucleotide polymorphisms (SNPs) in PfCRT in field isolates correlate with a resistance phenotype in *in vitro* assays and are sensitive markers for treatment failure in patients^8^,^9^. However, these molecular markers are not always specific because other variables such as previous exposure to malaria can influence treatment response in patients^10^.

One polymorphism at position 76 (K76T) in the first TMD of PfCRT seems to be key to CQ resistance. This substitution removes a positive charge from a predicted substrate-binding site in PfCRT, allowing protonated CQ to escape from the FV down its electrochemical gradient^11^. Other mutations (K76I and K76N) in this position also arise when *P. falciparum* is exposed *in vitro* to lethal concentrations of CQ, allowing parasites to survive and supporting the critical role of this residue^1^,^6^.

The native function of PfCRT is not clear, although it has been postulated to be involved in hemoglobin catabolism, possibly by mediating the transport of hemoglobin-derived peptides/amino acids from the FV^12^, a hypothesis consistent with recent heterologous expression and metabolomics studies^7^,^13^,^14^. PfCRT has also been proposed to function as a chloride channel, a proton pump or a regulator of proton pumps, a general activator or modulator of transport systems (reviewed in^11^) or, most recently, a proton-coupled transporter of a broad range of cationic substrates^15^. There are many reasons to elucidate the function of PfCRT in parasites, including the suggestion that PfCRT could itself become a new drug target^16^,^17^, or that chemosensitizing agents could be directed against PfCRT to restore the efficacy of CQ^16^,^17^,^18^. Furthermore, CQ continues to be used in the treatment of non-falciparum malarias. It may also regain efficacy against falciparum malaria in areas where usage has been tightly regulated, since the withdrawal of CQ can result in dramatic decreases in the prevalence of CQ-resistant parasites^19^.

Here, mutations in *pfcrt* that alter parasite phenotype give new insights into its native function as a transporter. The novel and pleiotropic phenotypic characteristics associated with mutated PfCRT include altered FV morphology and changes in quinoline sensitivities. We also investigated the effect of these changes on the parasite’s sensitivity to other antimalarial classes, such as the artemisinins, that some have considered to act (at least in part) in the FV of the parasite^20^,^21^.

## Results

### New and previously described mutations in *pfcrt*

**Figure 1:**
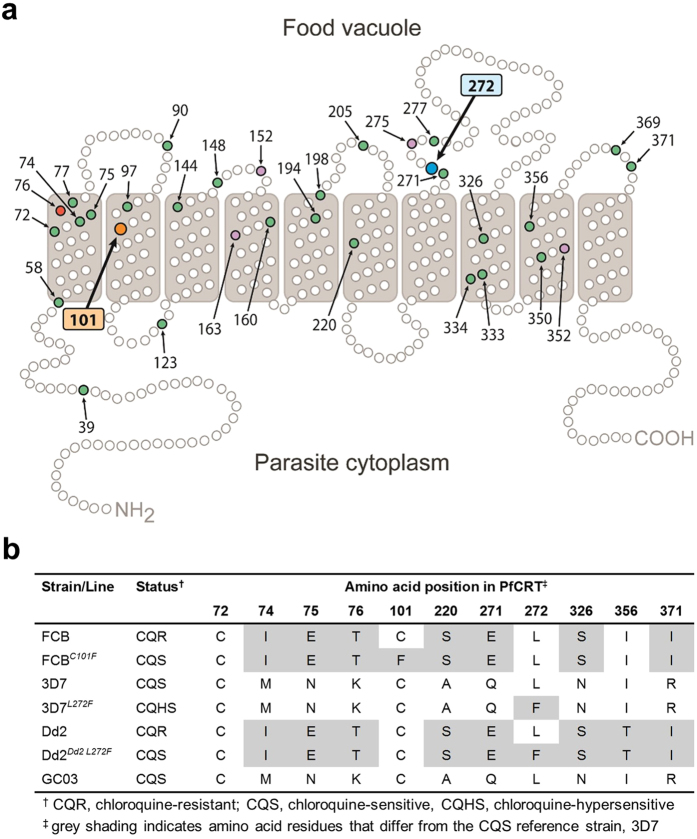
PfCRT mutations. (**a**) Schematic representation of PfCRT and positions of previously identified polymorphisms^8^,^71^ from field isolates (green circles) and from drug-pressured laboratory lines (purple circles). The critical CQ resistance mutation site (K76) is shaded red, and the two residues at which mutations are described in this study are shaded in orange (C101) and blue (L272). (**b**) PfCRT haplotypes included this study.

SNPs were identified in *pfcrt* in two different *P. falciparum* lines (Fig. 1a,b). The first was discovered after isolating amantadine (AMT)-resistant mutants of the CQ-resistant parasite strain FCB, following selection with 80 μM of this antiviral agent. Viable parasites were observed in one of four drug-pressured flasks at 42 days, whereas none had emerged within the remaining flasks by 60 days. PCR amplification and sequencing of *pfcrt* in four clonal lines derived from the AMT-resistant culture detected a single non-synonymous SNP, g302t. This encoded the amino acid mutation C101F. These lines were therefore designated FCB^*C101F*^. Position 101 is predicted to lie within the second TMD of PfCRT (Fig. 1a). This mutation was earlier observed in a CQ-resistant Dd2 parasite line derived by continuous piperaquine (PPQ) pressure^22^, although that study did not describe any changes in parasite morphology.

The second parasite line, derived from the CQ-sensitive strain 3D7, was selected by blasticidin (BSD) pressure as an inadvertent outcome of transfection experiments on an unrelated gene (that had aimed to achieve single cross-over homologous recombination with a tagging plasmid under BSD selection)^23^. After several weeks of selection, *pfcrt* cDNA transcripts of the daughter parasite line and parental 3D7 were sequenced. A mutation at position c814t in the *pfcrt* coding sequence, resulting in the amino acid mutation L272F, was detected in the selected line, designated 3D7^*L272F*^, and was absent in its parent. This substitution is positioned immediately after the seventh predicted TMD, placing it in the FV compartment (Fig. 1a). To our knowledge, this mutation has not been reported previously. No other mutations in *pfcrt* were detected in either of the new parasite lines.

Given that 3D7^*L272F*^ arose in unusual circumstances (BSD is a general inhibitor of protein translation and is not thought to target the FV), whole-genome sequencing was undertaken to identify further mutations. This confirmed the presence of the c814t mutation in *pfcrt* and identified only 2 additional SNPs. The first was c5549g in PF3D7_1229100 (the *P. falciparum* multidrug resistance-associated protein 2, PfMRP2), resulting in a stop-gain mutation (S1850*) and the loss of 259 amino acids from the C-terminus. The second was t1032a in PF3D7_1462400 (a conserved protein of unknown function), resulting in a stop-gain mutation (Y344*) and the loss of 2979 amino acids from the C-terminus. Truncation of the latter sequence has been observed in other laboratory clones of 3D7^24^. Furthermore, there was no evidence of integrated copies of the plasmid vector containing the BSD selection marker^23^, which had been used during the generation of the 3D7^*L272F*^ line.

### Enlarged FVs of parasites with mutations in *pfcrt*

**Figure 2:**
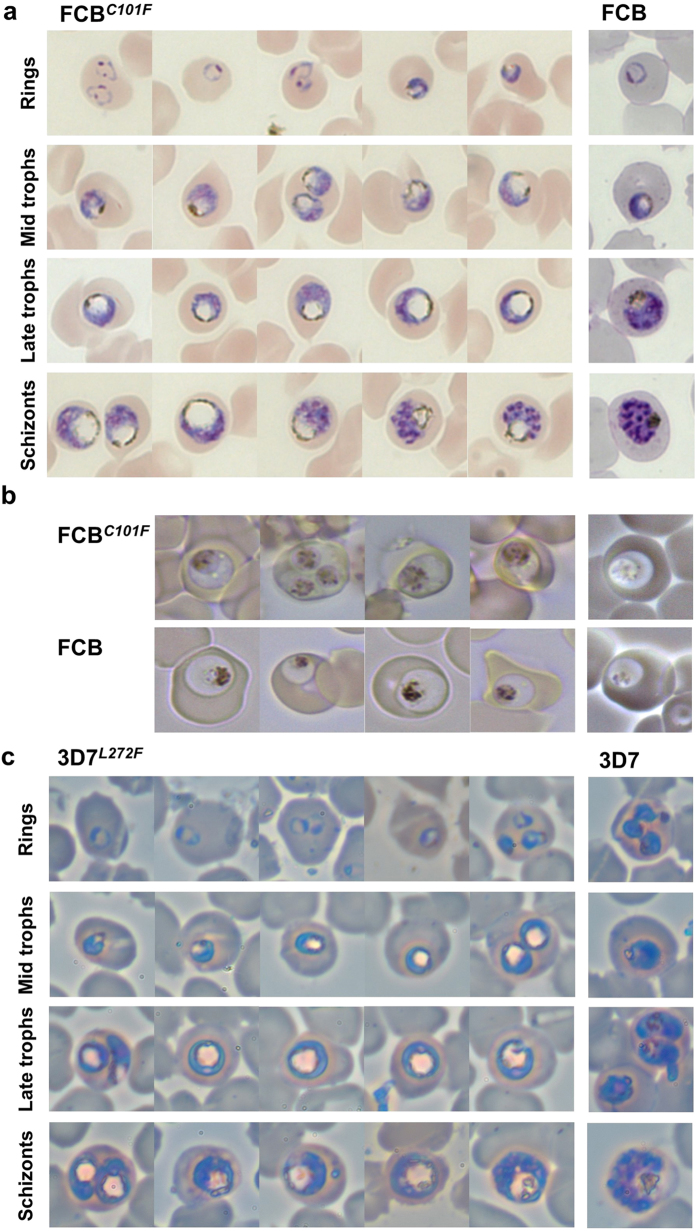
Representative morphology of parasite lines FCB^*C101F*^ and 3D7^*L272F*^. (**a**) Appearance of enlarged FVs in fixed FCB^*C101F*^ parasites (*left panel*), when compared with parental FCB parasites of similar developmental stages (*right panel*). (**b**) Images of live FCB^*C101F*^ and FCB trophozoite-stage parasites, using bright-field and dark-field microscopy (*left* and *right panels*, respectively). (**c**) Appearance of enlarged FVs in fixed 3D7^*L272F*^ parasites (*left* panel), when compared with parental 3D7 parasites of similar developmental stages (*right panel*). The diameter of a RBC is ~7 μm.

**Figure 3:**
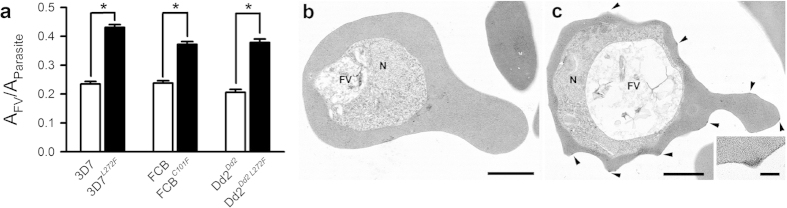
Morphological comparisons of parasites with mutations in *pfcrt* compared with their parental controls. (**a**) Parasites were synchronized by sorbitol lysis and areas of FVs and parasites (approximately 38 h post-invasion) were measured and expressed as ratios (A_FV_/A_Parasite_). 3D7, FCB and Dd2^Dd2^ (open bars; n = 72, 31 and 42, respectively) and 3D7^*L272F*^, FCB^C101F^ and Dd2^Dd2 L272F^ (closed bars; n = 84, 58 and 88, respectively) parasites were analyzed and significant enlargement of the FV was confirmed in 3D7^*L272F*^, FCB^*C101F*^ and Dd2^*Dd2 L272F*^ parasites relative to 3D7, FCB and Dd2^*Dd2*^, respectively (*p < 0.0001: two-tailed, unpaired, Student’s *t*-test). (**b**,**c**) Transmission electron micrographs of 3D7 and 3D7^*L272F*^ parasites, respectively, showing the food vacuole (FV) and nucleus (N). Note the enlarged electron lucent FV in 3D7^*L272F*^ (suggesting changes in the process of hemoglobin degradation and formation of hemozoin crystals). RBCs infected with 3D7^*L272F*^ displayed approximately 7.5-fold more knobs (arrowheads) on the host surface than 3D7-infected RBCs, although this is likely due to sub-population selection rather than a direct link to the mutation in *pfcrt*. (**insert**) Detail of a knob. Bars represent 1 μm (**b**,**c**) and 100 nm (**insert**).

A monstrously swollen FV was observed at all stages that ordinarily display a vacuole in the asexual cycle of both parasite lines FCB^*C101F*^ and 3D7^*L272F*^ (Fig. 2). This phenotype was stably maintained in the parasites following repeated rounds of parasite culture and cryopreservation. The enlarged FVs were already apparent in the early to mid trophozoite stages of the FCB^*C101F*^ line, when compared with FVs from FCB parental controls (Fig. 2a left and right panels). In more mature FCB^*C101F*^ parasites, the FVs were strikingly clear in appearance, with hemozoin crystals apparently marginalized to the FV periphery and opposite the developing nuclei, although live imaging suggests that the hemozoin is distributed normally (Fig. 2b). The immature ring stages of development were indistinguishable from those of the parental strain. Similar findings were evident in the parasite line 3D7^*L272F*^ when compared with 3D7 (Fig. 2c left and right panels). Measurement of the area of the FV was also undertaken and expressed as a ratio of the parasite’s area to correct for parasite age (Fig. 3a). This confirmed that FCB^*C101F*^ and 3D7^*L272F*^ parasites have a relative FV/parasite area that is approximately twice that of FCB and 3D7, respectively (*p* < 0.0001). Neither FCB^*C101F*^ nor 3D7^*L272F*^ parasites appeared to be enlarged within their host red blood cells (RBCs).

The 3D7^*L272F*^ line was selected for a more detailed characterization. The appearances of parasites examined with transmission electron microscopy (TEM) were consistent with observations made with light microscopy (Fig. 3b,c), with few differences evident between parental strains and daughter parasite lines except for the size of the FV. Specific to this line, TEM also revealed that “knobs”, electron dense protrusions of the RBC membrane caused by parasite infection, which are important determinants of cytoadherence^25^ and which are often lost from infected RBCs during long term parasite culture^26^, were displayed approximately 7.5-fold more on the host surface of 3D7^*L272F*^-infected RBCs than 3D7-infected RBCs. This is unlikely to be directly related to the mutation in *pfcrt* and may be due to sub-population selection.

Since BSD pressure has been shown to alter infected RBC permeability^27^,^28^,^29^, electrophysiological transport studies were also undertaken to compare 3D7 and 3D7^*L272F*^-infected RBCs, although no differences were observed (Supplementary Fig. S1).

### *In vitro* sensitivity to antimalarials

Both cell lines with mutations in *pfcrt* displayed altered susceptibility to antimalarials when compared with the parental strains (Table 1). Using a 72 h *in vitro* growth inhibition assay that yields IC_50_ values, FCB^*C101F*^ parasites were found to be 83 fold less susceptible to AMT (used in its selection). FCB^*C101F*^ showed a 5–6 fold increase in sensitivity to CQ, yet interestingly still retained the characteristic verapamil (VP)-reversibility of CQ-resistant parasites^30^. Furthermore, compared with FCB, FCB^*C101F*^ was significantly (*p* < 0.01) more sensitive to quinolines (quinine (QN), quinidine (QD) and monodesethyl amodiaquine (MDAQ)) but not the arylmethanol, mefloquine (MQ). There was a small (29%) increase in sensitivity to artemisinin (ART; *p* <0.01). The FCB^*C101F*^ line became approximately 2-fold more resistant to PPQ relative to controls (*p* < 0.05).

In similar experiments, 3D7^*L272F*^ parasites, assayed over 48 h *in vitro,* were ~2.5 fold more sensitive to CQ than 3D7, with respective IC_50_ values of 6.1 and 15 nM (*p* < 0.01). The 3D7^*L272F*^ parasites were also slightly more sensitive to QN than 3D7 parasites. VP sensitivity was not examined because unlike FCB, the 3D7 line is already CQ-sensitive. The increased sensitivity to CQ therefore indicates that 3D7^*L272F*^ is a ‘CQ-hypersensitive’ parasite line. The mean IC_50_ values for MQ, MDAQ, PPQ and ART were similar between the 3D7^*L272F*^ and 3D7 parasites (Table 1).

### Transfection studies

**Figure 4:**
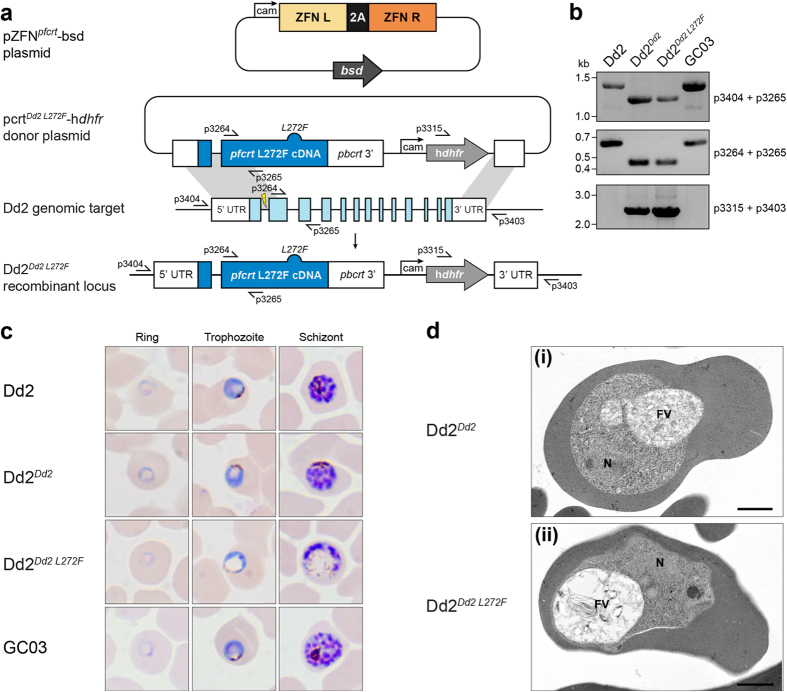
Introduction of PfCRT L272F into Dd2. (**a**) Schematic of zinc-finger nuclease (ZFN)-mediated generation of vacuole-enlarged parasites. Dd2 parasites were first enriched for the episomal pcrt^*Dd2 L272F*^-h*dhfr* or pcrt^*Dd2*^-h*dhfr* donor plasmids (latter not shown). The donor plasmids encoded a cDNA copy of the Dd2 *pfcrt* allele (dark blue, plasmid), either L272F-mutated (dark blue bump) or wild-type (not shown), followed by a *dhfr* selection cassette (light grey). Each donor-enriched parasite was then transfected with the pZFN^*pfcrt*^-bsd plasmid, expressing the genomic (light blue) *pfcrt* intron 1-targeting ZFN pair (ZFN L and ZFN R, orange) and the *bsd* selection cassette (dark grey). ZFN-induced recombination in *pfcrt* yielded either control Dd2^*Dd2*^ (not shown) or Dd2^*Dd2 L272F*^ parasites (dark blue, locus). (**b**) PCR verification of parental, recombinant control, and Dd2^*Dd2 L272F*^ parasite clones. Primer (p) positions are shown in *panel a*. (**c**) Light mi**c**roscope analysis of representative examples of parental, recombinant control, and experimental parasite clones. Ring morphology for each parasite Dd2, Dd2^*Dd2*^, Dd2^*Dd2 L272F*^, and GC03 was normal. Progression through the trophozoite and schizont stages showed normal morphological development except for the Dd2^*Dd2 L272F*^ clone, which exhibited the characteristic enlarged vacuole and diffuse hemozoin phenotypes seen in 3D7^*L272F*^ and FCB^*C101F*^ Giemsa stained parasites. (**d**) Transmission electron micrographs of Dd2^*Dd2*^ (i) and Dd2^*Dd2 L272F*^ parasites (ii) showing similar cytoplasmic appearances except for the enlarged food vacuole (FV) in Dd2^*Dd2 L272F*^. Note neither parasite exhibits knobs. This confirms similar morphological appearances to those of 3D7 and 3D7^*L272F*^, respectively but without knob formation. N—Nucleus. Bars represent 1 μm.

To confirm the phenotype observed in 3D7^*L272F*^, we engineered the L272F mutation in *pfcrt* using zinc-finger nuclease mediated allelic replacement^31^ in the Dd2 line and compared results with congenic controls. Figure 4a,b illustrate this strategy and provide confirmation of integration. As observed in 3D7^*L272F*^, significant FV distension (~2 fold as measured by vacuolar area relative to parasite area; Fig. 3a) was generated by introduction of this single amino acid change (Fig. 4c,d). However, a significant increase in BSD resistance was not observed between the Dd2^*Dd2 L272F*^ line and its congenic control, Dd2^*Dd2*^ (Table 2), which suggests that the PfCRT L272F mutation was not primarily responsible for the BSD resistance found in 3D7^*L272F*^ parasites. The parental strain Dd2 and the congenic control Dd2^*Dd2*^ were both CQ-resistant. However, Dd2^*Dd2 L272F*^ was considerably more susceptible to CQ and monodesethyl chloroquine (MDCQ) than the Dd2^*Dd2*^ line, although the IC_50_ values of the L272F mutant remained higher than the fully CQ-sensitive reference line GC03 (Table 2). ART sensitivity, as measured in these IC_50_ assays, was unaltered across parasites. There were no differences in whole-cell electrophysiological properties between the Dd2^*Dd2*^ and Dd2^*Dd2 L272F*^ parasite lines (Supplementary Fig. S1) and the RBCs infected with Dd2^*Dd2 L272F*^ parasites remained knobless (Fig. 4d), suggesting that the increased expression of knobs in 3D7^*L272F*^-infected RBCs was not related to the L272F mutation in *pfcrt*.

### Measurements of CQ transport via the C101F and L272F variants of PfCRT

The *Xenopus* oocyte system for the heterologous expression of PfCRT^7^ was employed to investigate the effect of the C101F and L272F mutations on the ability of PfCRT to mediate CQ transport. The L272F and C101F mutations were introduced into the Dd2 haplotype of PfCRT (PfCRT^Dd2^, from the CQ-resistant strain Dd2; Fig. 1b) and L272F was also introduced into PfCRT^3D7^ (from the CQ-sensitive strain 3D7; Fig. 1b). The resulting variants (L272F PfCRT^Dd2^, L272F PfCRT^3D7^, and C101F PfCRT^Dd2^), as well as PfCRT^Dd2^ and PfCRT^3D7^, were expressed in oocytes. Localization of each of the PfCRT variants to the oocyte plasma membrane was confirmed by immunofluorescence assay (Supplementary Fig. S2a) and a semiquantitative western blot analysis^32^ indicated that the different PfCRT proteins were present at similar levels in the oocyte membrane (Supplementary Fig. S2b). The ability of the PfCRT variants to mediate [^3^H]CQ transport was measured in an acidic medium (pH 5.5), in which the majority of CQ is protonated. The extent to which oocytes expressing PfCRT^Dd2^ accumulate [^3^H]CQ varies considerably between batches of oocytes from different frogs, with the PfCRT^Dd2^-expressing oocytes accumulating between 8 and 45 times more [^3^H]CQ than the control (non-injected and PfCRT^3D7^-expressing) oocytes. Hence, within each experiment uptake was expressed relative to that obtained for oocytes expressing PfCRT^Dd2^ (in the absence of inhibitors). Non-injected oocytes and oocytes expressing PfCRT^3D7^ have previously been shown to take up CQ to similar (low) levels via simple diffusion of the neutral species of the drug^7^,^32^; this represents the ‘background’ level of CQ accumulation in oocytes, which in this study was estimated by measuring CQ uptake into PfCRT^3D7^-expressing oocytes (see Supplementary Fig. S3).

**Figure 5:**
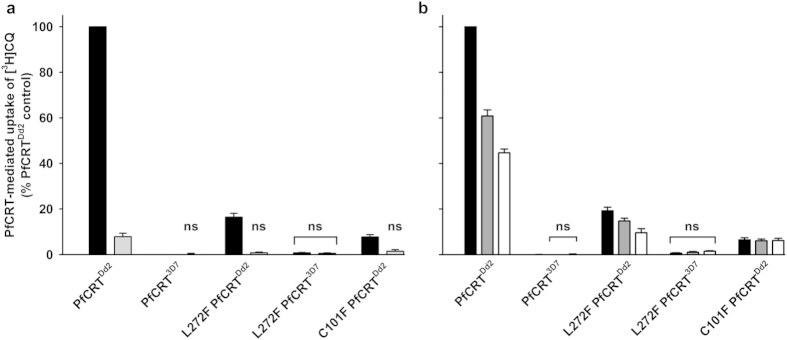
CQ transport activity of the C101F and L272F variants of PfCRT in *Xenopus* oocytes. (**a**,**b**) The upt**a**ke of [^3^H]CQ into oocytes expressing PfCRT was measured in the absence (closed bars) or presence of 250 μM VP (light grey bars; **a**), 100 μM BSD (dark grey bars; **b**), or 500 μM BSD (open bars; **b**). Within each experiment, measurements were made from 10 oocytes per treatment and uptake was expressed relative to that measured in the PfCRT^Dd2^-expressing oocytes under control conditions. The normalized data obtained from 4–5 separate experiments (each using oocytes from different frogs) were then averaged and are shown + SEM. Both panels show PfCRT-mediated CQ uptake, calculated by subtracting CQ uptake measured in PfCRT^*3D7*^-expressing oocytes (*i.e.* the component of CQ accumulation attributable to diffusion; see Supplementary Fig. S3) from that measured in oocytes expressing a variant of PfCRT. In the control treatments, the rates of CQ uptake (pmol/oocyte/h; n = 9 ± SEM) in oocytes expressing PfCRT^Dd2^ and PfCRT^3D7^ were 23.6 ± 2.3 and 1.3 ± 0.2, respectively. ‘ns’ denotes no significant difference (*p* > 0.05) in CQ accumulation between oocytes expressing a PfCRT variant (in the presence or absence of VP or BSD) and that measured in the PfCRT^3D7^-expressing oocytes under control conditions.

In the data presented in Fig. 5a,b, oocytes expressing PfCRT^Dd2^ showed an 11 to 40-fold (mean and SEM of 21 ± 3; n = 9 separate experiments) increase in CQ uptake relative to the PfCRT^3D7^-expressing control. The component of CQ accumulation attributable to diffusion (*i.e.* the uptake of CQ measured in PfCRT^3D7^-expressing oocytes) was subtracted to obtain the PfCRT-mediated component of CQ transport. Supplementary Figure S3 shows the total level of CQ accumulation in each oocyte and treatment type. The introduction of L272F or C101F into PfCRT^Dd2^ substantially reduced the protein’s ability to transport CQ (by ~82% and ~93%, respectively; *p* < 0.001, ANOVA) whereas the introduction of L272F into PfCRT^3D7^ was without effect (*p* > 0.05). The addition of the CQ resistance-reverser VP (250 μM) reduced PfCRT^Dd2^-mediated CQ transport by ~93% (*p* < 0.001) and also dramatically decreased CQ uptake via L272F PfCRT^Dd2^ and C101F PfCRT^Dd2^ (by ~84% and ~92%, respectively; *p* < 0.01), such that the accumulation of CQ in the latter two treatments was not significantly different from that measured in the PfCRT^3D7^-expressing controls (*p* > 0.05).

To investigate how BSD pressure might have produced the 3D7^*L272F*^ mutant, interactions between the PfCRT variants and BSD were assessed by measuring the uptake of [^3^H]CQ in the presence of unlabeled BSD (100 or 500 μM; Fig. 5b). The addition of BSD reduced CQ transport via PfCRT^Dd2^ by ~39% (100 μM; *p* < 0.001) and ~56% (500 μM; *p* < 0.001) and, to a lesser degree, decreased CQ uptake via L272F PfCRT^Dd2^ (by ~22% (*p* > 0.05) and ~49% (*p* < 0.01), respectively). Neither concentration of BSD reduced the C101F PfCRT^Dd2^-mediated transport of CQ (*p* > 0.05), nor was the accumulation of CQ in the PfCRT^3D7^-expressing controls affected (*p* > 0.05). Note that the micromolar concentrations of the compounds used here to inhibit PfCRT are physiologically relevant given that when present in the extracellular solution at nanomolar levels, these protonatable drugs are expected to accumulate within the parasite’s FV via weak-base trapping to micromolar or millimolar concentrations.

## Discussion

Mannaberg stained parasites with Romanowsky’s dyes and published detailed studies on the effects of QN against *P. falciparum,* which described the emergence of a ‘dropsical distension’ (enlarged FV) in mature parasites^33^. Here, we describe a similar peculiar phenotype of *P. falciparum* parasites that is visible without the application of antimalarial drugs. This phenotype is comparable between two parasite lines that have mutations in *pfcrt* in different positions (amino acids 101 and 272) and that have been selected by two chemically unrelated compounds (AMT and BSD). These mutations confirm that *pfcrt* encodes a function that is critical to maintaining FV volume. In support of this function, mutations in PfCRT that cause CQ resistance have been reported to increase FV volume^34^. However, the parasite lines described in this present study have clearly enlarged FVs but with PfCRT mutations that render the parasites more CQ sensitive than their control strains (be that either CQ-sensitive 3D7 or CQ-resistant FCB), suggesting an alternative mechanism of FV volume regulation is induced.

**Figure 6:**
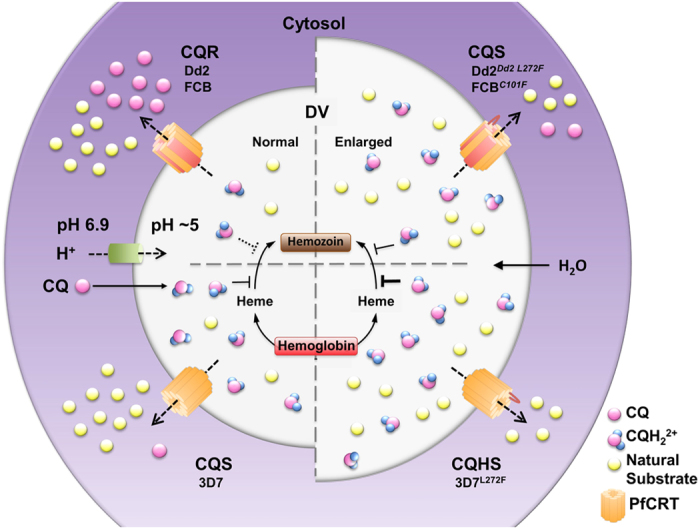
Hypothetical schematic model of the effects of PfCRT mutations. The FV is acidified by a vacuolar proton pump to create a suitable environment for hemoglobin digestion. The acidic nature of the FV also leads to near complete diprotonation of CQ, which diffuses across the FV membrane in an uncharged form (CQ) and accumulates as a charged form (either CQH^+^ or CQH_2_^2+^, although predominantly CQH_2_^2+^). CQH_2_^2+^ interferes with the polymerization of toxic heme to non-toxic hemozoin, which leads to parasite death. In normal CQ-sensitive (CQS) parasites, PfCRT, which contains a positive charge in its pore (K76), exports its natural substrates but little, if any, CQH_2_^2+^. Thus, CQH_2_^2+^ accumulates in the FV and causes parasite death. In CQ-resistant (CQR) parasites, the positive change in the pore of PfCRT is lost (K76T) and both its natural substrates and CQH_2_^2+^ are transported out of the FV. As CQH_2_^2+^ cannot accumulate in the FV, the parasites become resistant to the drug. In 3D7^*L272F*^ parasites (where the parent strain is already CQS), the mutation may reduce residual transport of CQH_2_^2+^ out of the FV even further or completely, leading to a greater FV accumulation of CQH_2_^2+^ and CQ-hypersensitivity or some other mechanism may be responsible for this phenomenon. The mutation also leads to a reduction in the export of natural substrates, resulting in a build-up of these substrates. This causes water to enter the FV by the process of osmosis, leading to swelling. In FCB^*C101F*^ and Dd2^*Dd2 L272F*^ parasites (where the parent strains are CQR), the mutations reduce the export of CQH_2_^2+^ back towards levels measured in CQS lines and also reduce natural substrate export, leading to normal CQ sensitivity and FV swelling, respectively. Note mutations in PfCRT (orange graphic) are denoted by red transmembrane or loop regions, depending on the location of the amino acid change (see Fig. 1a).

An enlarged FV is also often observed in the presence of protease inhibitors, such as E64 or leupeptin^35^. Interference with the digestion of hemoglobin leads to a buildup of darkly staining FVs in electron micrographs and, eventually, to parasite death. The parasites described here have enlarged FVs but these are electron lucent (Figs 2 and 4), suggesting that the digestion of hemoglobin is relatively unaffected (further supported by the presence of visible hemozin within the FVs). The simplest explanation for these observations is that the C101F and L272F mutations interfere with the transport of the natural substrates of PfCRT out of the FV. The resulting increase in FV osmotic pressure would lead to water ingress and produce the unusual swelling observed in the FV of the FCB^*C101F*^, 3D7^*L272F*^, and Dd2^*Dd2 L272F*^ parasites. Figure 6 presents a schematic model of this process. These morphological changes are associated with other phenotypic changes (which are discussed below). The natural substrate(s) of PfCRT are yet to be identified. Studies performed with other PfCRT expression systems have reported that the protein might function as a chloride channel, a proton pump, an activator of Na^+^/H^+^ exchangers and non-specific cation channels or, most recently, a transporter of cationic amino acids as well as a very broad range of other cations^15^. However, in many of these studies the insertion of PfCRT into the foreign membrane required its fusion to other proteins/polypeptides, and in the most recent study the additions to PfCRT were at both the N- and C- termini, almost doubled its size, and included a protein of undetermined function^15^. Moreover, in this and the previous studies, little or no interaction could be detected between PfCRT^Dd2^ and known inhibitors of this protein (*e.g.* VP). Of significant note, the transport kinetics for the proposed natural substrates did not differ significantly between PfCRT^Dd2^ and PfCRT^3D7^—despite multiple lines of evidence indicating that PfCRT^Dd2^ imparts a substantial fitness cost^13^,^36^,^37^,^38^. Furthermore, the recent finding that much higher levels of acidic amino acids and/or short acidic peptides accumulate within CQ-resistant parasites than in CQ-sensitive strains^7^,^13^,^14^ is not readily reconciled with PfCRT functioning as a chloride channel, a proton pump, or a non-specific cation channel/transporter. These, plus other inconsistencies in the data, suggest that PfCRT does not function correctly when fused to other proteins and that the natural function of PfCRT remains to be resolved.

AMT is an antiviral agent with moderate antimalarial activity that is more potent against CQ-resistant parasites than against CQ-sensitive strains^39^. AMT is likely to accumulate in the FV via weak-base trapping^40^ and is a low-affinity inhibitor of the PfCRT^Dd2^-mediated transport of CQ in the oocyte system^7^. While the antiplasmodial target of AMT remains unclear, AMT resistance has been linked previously to novel PfCRT mutations (S163R, I356V and V369F; Fig. 1) selected in parasites harboring CQ resistance-associated alleles of *pfcrt*; these mutations were linked with the loss of CQ resistance in the AMT-resistant mutants^41^,^42^. Here, a different single mutation (C101F) in the CQ-resistant FCB strain was likewise associated with a gain of AMT resistance and a reduction in CQ resistance. This mutation was identified previously in a PPQ-pressured parasite line that appeared to have acquired an unstable PPQ resistance phenotype via multiple genetic changes^22^. One of two PPQ-revertant lines derived during that study was ~2-fold more resistant to PPQ than the parental Dd2 strain, which along with a reduction in CQ resistance, is consistent with the data reported here for FCB^*C101F*^.

It has been suggested that the S163R mutation reintroduces a positive charge into the PfCRT binding pocket/translocation pore, thereby compensating for the loss of the positively-charged lysine residue from position 76^11^ and resulting in a dramatic reduction in the ability of the protein to transport protonated CQ^7^. The S163R mutation also abolishes the CQ resistance-reversing effect of VP^42^. The C101F and V369F mutations both entail the introduction of a bulky hydrophobic residue, rather than one carrying a positive charge, and it is interesting to note that VP still exerted a resistance-reversing effect in the FCB^*C101F*^ parasites (Table 1)—even though they were considerably less resistant than the FCB strain to CQ. These observations are consistent with our direct measurements of CQ transport via C101F PfCRT^Dd2^ (Fig. 5), which confirmed that this protein possesses a relatively low level of CQ transport activity that can be inhibited by VP. Likewise, our finding that the introduction of L272F into PfCRT^Dd2^ causes a dramatic (but not complete) reduction in the protein’s capacity for CQ transport (Fig. 5) correlates well with the low level of CQ resistance exhibited by the Dd2^*Dd2 L272F*^ line. The phenylalanine residues are likely to be proximate to the binding site and/or translocation pore of PfCRT (Fig. 1) where their bulky side chains may act to significantly hinder the transport of certain drugs out of the FV, including CQ, QN and QD (based on the growth assay data presented in Tables 1 and 2). Another mutation that has arisen under the AMT pressure of a CQ-resistant strain, and which also resulted in both the introduction of a phenylalanine residue (V369F) and a significant reduction in CQ resistance, did not cause the FV to swell^41^. Hence, the enlarged FV phenotype described here appears to manifest only when the bulky phenylalanine side chain is inserted at specific positions within PfCRT.

BSD is used commercially as a fungicide against a rice blast disease and acts by inhibiting protein translation. In biological research it is used to select transformed cells. BSD resistance has previously been linked to altered expression of *clag3.1* and a decrease in the RBC membrane permeability mediated by the new permeability pathways, NPP^28^,^29^. Neither 3D7^*L272F*^ nor Dd2^*Dd2 L272F*^ parasites differed in their electrophysiological NPP characteristics when assayed by whole-cell patch-clamp methods (Supplementary Fig. S1). This suggests that one or more *clag3.1*-independent BSD resistance mechanisms exist. Our results indicate that, under the conditions of the growth assay, the L272F mutation does not cause a significant increase in BSD resistance when introduced in isolation into Dd2 parasites (Table 3). Nevertheless, BSD was found to inhibit CQ uptake via PfCRT^Dd2^ and the potency of this interaction appeared to decrease upon the introduction of L272F (the addition of 100 μM BSD was more effective against PfCRT^Dd2^ than against L272F PfCRT^Dd2^; Fig. 5). A demonstration that BSD interacts with PfCRT^Dd2^, and to a lesser extent with L272F PfCRT^Dd2^, provides support for the idea that BSD also interacts with, and may be transported by, PfCRT^3D7^. BSD contains two protonatable nitrogens with pK_a_ values that are well above 7. It is therefore likely to be accumulated within the FV via weak-base trapping to high micromolar, or even millimolar, concentrations when present in the extracellular solution at the concentration (5.4 μM) under which the 3D7^*L272F*^ line arose. Given that BSD inhibits protein translation, which occurs outside of the FV, it is possible that a PfCRT^3D7^-mediated efflux of BSD from the FV could increase the drug’s access to its main target and that the L272F mutation diminishes this activity, such that the drug remains sequestered within the FV. The finding that L272F PfCRT^Dd2^ does not confer BSD resistance when expressed in Dd2 parasites suggests that either (1) PfCRT^Dd2^ is already a poor transporter of BSD (noting that the 3D7 and Dd2 haplotypes of PfCRT differ by eight mutations) and a reduction in this meager transport activity by the introduction of L272F has little effect on the accumulation of BSD within the FV, or (2) PfCRT^Dd2^ has a very high capacity for BSD transport, such that the presence of L272F causes only a modest reduction in its ability to redistribute BSD from the FV into the cytosol. In any case, it is clear that if L272F is directly involved in altering the parasite’s susceptibility to BSD, its effect is only evident when one or more other changes are present. In this regard, it is worth noting that one of the two mutations identified by whole genome analysis of the 3D7^*L272F*^ line would result in a truncated PfMRP2 protein. An understanding of the contribution of this transporter to BSD susceptibility, and its possible interplay with the BSD transport activity of PfCRT, requires further transfection-based analysis.

A diverse range of PfCRT variants implicated in conferring CQ resistance have been shown to exhibit CQ transport activity (to varying extents) in the oocyte system^7^,^32^. However, CQ transport via the wild-type form of the protein (found in CQ-sensitive parasites such as 3D7) has not been detected in this assay. Although it is possible that a very low level of CQ efflux is mediated by PfCRT^3D7^ *in situ*, and that the introduction of the L272F mutation abolishes this activity, it is perhaps unlikely that this would result in the 2-fold difference in the CQ IC_50_ observed between the 3D7^*L272F*^ and 3D7 parasites.

An alternative explanation for the hypersensitivity of the 3D7^*L272F*^ line to CQ and QN entails viewing the effect of the L272F mutation as being equivalent to the effect of an ‘anti-PfCRT’ drug. The presence of the L272F mutation causes the FV to swell, probably because it significantly obstructs the PfCRT-mediated efflux of solutes from this compartment. A drug that binds to the substrate-binding site of PfCRT^3D7^, thereby blocking or dramatically reducing its normal activity, would achieve a similar effect. If such an anti-PfCRT drug were applied in combination with CQ or QN, which also exert their antimalarial effects in the FV, it is possible that an additive, or even synergistic, interaction would be observed in 3D7^*L272F*^ parasites. That is, the 3D7^*L272F*^ parasites have a dis-functional FV and this could render certain drugs more effective against them; perhaps the altered composition of the FV lumen alters the solubilities of CQ and QN and/or their affinities for heme. It is not immediately apparent why AQ, MDAQ, and PPQ are not likewise more active against 3D7^*L272F*^ parasites. However, it is worth noting that AQ, MDAQ and PPQ are much more lipophilic than CQ and this may explain differences in potency^43^,^44^. Hence, the antimalarial activities of the latter drugs might be less sensitive to changes in FV volume and composition. Alternatively, or in addition, it is also possible that extending the growth assays to 72 or 96 h (from 48 h) would reveal differences in AQ, MDAQ, and PPQ sensitivity between the 3D7 and 3D7^*L272F*^ parasites.

If artemisinins act mainly in the FV, a disputed suggestion^45^ (along with reports of predominantly non-FV-localized artemisinin targets, e.g.^46^,^47^), then their activities may also differ between the parental and mutant lines. There was no significant change in the ART sensitivity of the 3D7, Dd2 and GC03 lines and only a <30% change in the IC_50_ value obtained for FCB (which displays an IC_50_ value <15 nM). Recent observations made in a *P. berghei* model using protease knockouts that alter vacuolar morphology also leave artemisinin sensitivity unaltered^48^. This is also consistent with the lack of PfCRT expression in early ring stages^49^, when artemisinins exert their major antimalarial action *in vivo*. Similarly, the MF IC_50_ value was unaffected in the experiments presented here. Our observations therefore relate relatively large changes in the activities of several aminoquinolines to an enlarged FV phenotype that is caused by specific mutations in PfCRT.

Our results show for the first time that mutations at position 272 and 101 in PfCRT can hypersensitize parasites to CQ and enlarge the FV, thereby extending the function of this key transporter to include maintenance of FV morphology. We suggest that the introduction of a phenylalanine residue at either of these positions decreases the protein’s ability to transport its physiological substrate(s) (as well as certain drugs) and that the resulting build-up of the physiological substrate(s) causes the FV to swell. The fact that these mutations do not reintroduce a positive charge into the predicted binding cavity/translocation pore of PfCRT, as has been observed in other examples of laboratory parasites that revert to CQ-sensitive status (*e.g.*^42^), indicates that there is more than one type of single mutation—and therefore more than one mechanism—by which the CQ transport activity of PfCRT can be abrogated. This insight extends our understanding of the structure-function of PfCRT (*e.g.*^32^). Moreover, the finding that single mutations to the protein can result in gross changes to parasite morphology emphasizes the central role of this transporter in the physiological processes that occur within the FV and provides a novel insight into one of the factors constraining the evolution of PfCRT. The observation that BSD binds to, and appears to exert a selection pressure on, PfCRT further broadens the diversity of chemotypes that are known (or suspected) to interact with the protein. Our data encourage further studies to define agents that could reverse antimalarial drug resistance mediated by PfCRT by inhibiting its function.

## Methods

### Antimalarials and reagents

CQ, QN, QD, MQ, AQ, PPQ, ART, BSD, AMT and VP were purchased from Sigma Aldrich Chemical Co. MDAQ was purchased from Santa Cruz Biotechnology, Inc. MDCQ was a gift from William Ellis (Walter Reed Army Institute of Research, Silver Spring, MD). SYBR Green I was purchased from Invitrogen Corp. Drug stocks were prepared to 10 mM in DMSO or 70% ethanol and stored below -20 °C.

### *In vitro* culture and selection of parasites

*P. falciparum* 3D7 and 3D7^*L272F*^ parasites were cultured in human RBC suspensions using RPMI 1640 medium (Sigma-Aldrich; Cat. No. R0883-500ML) supplemented with 2 mM L-glutamine, 34 mM HEPES, 0.5% (*w/v*) Albumax I, 0.19 mM hypoxanthine, and 50 μg/ml gentamycin and maintained at 37 °C under 5% CO_2_. For parasite clone 3D7^L272F^, complete medium was supplemented with 2.5 μg/ml blasticidin-S HCl (Invitrogen). Parasite growth was followed by microscopic examination of Field’s stained thin blood smears. Synchronization of early trophozoite stages was achieved by incubating infected RBCs in 5% (*w/v*) sorbitol for 10 to 20 min at room temperature^50^. Following transfection studies^23^, parasites with abnormally enlarged FVs, as described in results, reappeared in culture under BSD pressure after four weeks. In order to select these parasites, the limiting dilution technique was used, and cloned parasites were identified by microscopy using thin blood smears^51^.

*P. falciparum* FCB and FCB^*C101F*^ parasites were cultured in AB^+^ or O^+^ human RBC suspensions using RPMI 1640 medium (Mediatech, Inc.) supplemented with 0.5% Albumax I, 29.8 mM sodium bicarbonate, 25 mM HEPES, 0.37 mM hypoxanthine, and 10 μg/ml gentamicin and maintained at 37 °C under an atmosphere of 90% N_2_, 5% CO_2_, and 5% O_2_. AMT-resistant *P. falciparum* was selected by single-step selection based on an earlier method described for CQ^6^. Before drug pressure, parasites of the FCB strain were grown to 5% mixed stage parasitemia at 5% hematocrit in 50 ml of media. This starter culture was then split equally into 4 flasks, with fresh media and RBCs to bring the volume in each flask to 50 ml and 5% hematocrit. When parasitemia of the 4 flasks had returned to 5%, the media was replaced with fresh media containing 80 μM AMT. At ~14 fold the IC_50_ value determined for FCB (Table 2), this concentration of AMT rapidly kills CQ-resistant parasites. For the first week after drug application, cultures were monitored daily by Giemsa-stained thin blood films. Fresh AMT-containing media changes were performed daily. At one week, 50% of the RBCs were replaced, and fresh AMT media was added. Cultures were then maintained every third day with fresh AMT media for the duration of the experiment and monitored by thin smear for emergent parasites. With every second media change, 50% of the RBCs were replaced with fresh cells. If no surviving parasites were observed after 60 days, the experiment was terminated. After 42 days, parasites were recovered from one of the 4 flasks, which were then cloned by limiting dilution^52^ in drug-free media. The mixed culture and four randomly chosen cloned lines were cryopreserved prior to DNA sequencing and drug susceptibility testing.

### Morphological measurements

For comparison, thin blood films of cultured parasite samples were made at various time points following sorbitol synchronization and stained with Field’s stain. Pictures were taken under the same conditions for the 3D7 (parent) strain and the 3D7^*L272F*^ line and analyzed with a Nikon Eclipse TE2000 inverted microscope. Areas were measured using ImageJ 1.44o software and the ratio was expressed as A_FV_/A_Parasite_. No image manipulations were carried out after recording.

For micrographs of FCB and FCB^*C101F*^, thin films from parasite cultures were stained with 2% (*v/v*) modified Giemsa (Karyomax®; Gibco) for 30 min. Slides were washed for 60 s in flowing distilled water, air-dried and mounted with coverslips. Images were photographed in bright field, using a Lexica DMI4000 inverted microscope under a 100X objective lens. Images were compiled in Adobe Photoshop CS5.1 and processed equally with a warming photo filter. Live parasite cultures were placed under coverslips and photographed under a 100X objective, using a Leica DM750 light microscope equipped with ICC50 HD digital camera. Images were adjusted for white balance with the Leica Application Suite software and cropped in Adobe Photoshop CS5.1.

For experiments with Dd2 parasites, thin blood smears were fixed with methanol, stained for 20 min in 10% (*v/v*) Giemsa (Invitrogen), washed, and air-dried. Images were taken with an Olympus DP12 digital camera attached to an Olympus CX 41 light microscope with a 100X objective (N.A 1.4x). Images were cropped and corrected for white balance using Adobe Lightroom 3.

### Electron microscopy

Samples of 3D7, 3D7^*L272*^, Dd2^*Dd2*^ and Dd2^*Dd2 L272F*^, synchronized at the mature trophozoite stage were fixed in 4% (*v/v*) glutaraldehyde in 0.1 M phosphate buffer and processed for routine electron microscopy, as described previously^53^. Samples were post fixed in osmium tetroxide, treated *en bloc* with uranyl acetate, dehydrated and embedded in Spurr’s epoxy resin. Thin sections were stained with uranyl acetate and lead citrate prior to examination in a JEOL1200EX electron microscope.

### *In vitro* inhibition assays

Sensitivity to CQ and other drugs for 3D7 and 3D7^*L272F*^ parasites was determined by measurement of [^3^H]-hypoxanthine incorporation over 48 h, as described previously^54^. Nine serial dilutions plus a control (no drug) were tested in quadruplicates and the experiment was repeated at least three times for each drug. The assay was performed always in parallel on 3D7 and 3D7^*L272F*^ parasites.

The *in vitro* susceptibility of FCB and the FCB^*C101F*^ line of *P. falciparum* to antimalarial drugs was measured in a 72 h, 96 well microplate fluorescence assay using SYBR Green I detection as described^55^,^56^. Drugs were serially diluted 2-fold in the microplates, except for AMT, which was diluted 3-fold. VP was used at a concentration of 0.8 μM where indicated. Synchronous (immature) ring-stage parasites were assayed at 0.2% parasitemia and 2% hematocrit. Assays were conducted every 48 h until three independent replicates were performed. For Dd2 parasites, the same methodology was used except parasites were also stained with 1.6 μM Mito Tracker Deep Red.

### Genotypic characterization of *pfcrt* gene

For 3D7 parasites, RNA was extracted from parasites collected in RNAlater, using QIAGEN RNeasy Mini Kit, and immediately used to retro-transcribe cDNA (QIAGEN, QuantiTect Rev. Transcription Kit). Mutation in *pfcrt* was investigated by PCR, as described previously^1^. The same primers (Supplementary Table S1), which amplified overlapping products, were used to sequence the products to cover the entire open reading frame (ORF) of the gene. Amplification of the gene and its sequencing was performed twice (by Beckman Coulter Genomics). Alignment of the reported 3D7 gene from PlasmoDB and 3D7 and 3D7^*L272F*^ sequenced genes was performed using MacVector software (version 11.1).

For FCB parasites, 4 clonal lines of FCB^*C101F*^ were used for *pfcrt* sequencing. All ORF sequences of *pfcrt* were amplified from *P. falciparum* genomic DNA^57^. PCR products were sequenced directly using an ABI 3730xl DNA analyzer (Applied Biosystems).

### Whole genome sequencing and variant detection

Genomic DNA was isolated and prepared from the parental *P. falciparum* parasite line 3D7 and 3D7^L272F^. A total of 10 μg of gDNA from each line was sheared to obtain a fragment size of ~200–400 bp using an E220 focused-ultrasonicator (Covaris) with the following settings: 10% duty cycle, intensity 5, 200 cycles per burst, 180 s treatment length. The resulting sheared gDNA was size selected on a 2% (*w/v*) low-melting agarose gel and then purified and concentrated using MinElute purification columns followed by the QIAquick PCR purification kit (QIAGEN). Barcoded libraries for Illumina TruSeq single-end sequencing were then constructed from the size-selected, sheared material using NEBNext DNA Library Preparation reagents (New England Biolabs) by following the standard Illumina (Illumina) library preparation protocol. Finally, barcoded libraries were size selected using Agencourt AMPure XP magnetic beads (Agencourt Biosciences, Beckman Coulter) thereby removing any adapter dimers and resulting in a highly enriched barcoded library of 200–400 bp adapter-ligated fragments. The quality of the final sequencing libraries was assessed using an Agilent 2100 Bioanalyzer (Agilent Technologies) run alongside the original size-selected fragmented gDNA from the same preparation, and the concentration of each library was quantified using a Quant-iT dsDNA Broad-Range Assay Kit (Invitrogen). The final libraries were multiplexed with three barcoded samples and 20% (*v/v*) PhiX control DNA (Illumina, Catalog # FC-110-3001) per lane and were sequenced using an Illumina HiSeq 2500 Rapid Run (150 bp) system (Illumina).

Sequencing outputs were uploaded into Galaxy^58^, which is hosted locally at the Millennium Science Complex at Pennsylvania State University. Sequence reads were mapped to the *P. falciparum* 3D7 reference genome v. 10.0 (http://plasmodb.org/common/downloads/release-10.0/Pfalciparum/) and pCam-BSD-PfATP6-doubleHA plasmid sequence^23^, using the Burrows-Wheeler alignment tool^59^, and files were converted to allow for further analysis (GATK/BAM-to-SAM)^60^. Sequence variations were detected by Freebayes (version 0.9.0.a) using stringent filtering parameters based on quality and read depth^61^,^62^. Then SNPeff (version 3.3) was applied to annotate and determine statistical significance of each variant^63^. Genome copy number variations were detected based upon local chromosomal read depth using CNVnator (version 0.3) and annotated with Intansv (version 0.99.3)^64^. Alignments and variants were visualized using the Integrative Genomics Viewer^65^. Unique reads were selected and filtered for Map Quality >30.

### Plasmid construction and generation of Dd2 recombinant parasites

The donor plasmid p*crt*^Dd2^-h*dhfr* has been previously reported^31^. The mutation-encoding plasmid, p*crt*^Dd2 L272F^-h*dhfr*, was generated by site-directed mutagenesis of p*crt*^Dd2^-h*dhfr*, using primers p3527 + p3528 (Supplementary Table S2).

ZFN-editing transfection methods have been previously described^31^. Briefly, Dd2 parasites were electroporated with either p*crt*^Dd2^-h*dhfr* or p*crt*^Dd2 L272F^-h*dhfr* donor plasmid^66^. On Day 1 post-electroporation, they were cultured in the presence of 2.5 nM WR99210 (obtained from Jacobus Pharmaceuticals Inc.). Once recovered, both p*crt*^Dd2^-h*dhfr* and p*crt*^Dd2 L272F^-h*dhfr* transfected parasites were electroporated a second time with pZFN^*crt*^-bsd separately. On Day 1 post-electroporation each transfection was cultured with 2 μg/ml blasticidin S (Invitrogen) and 2.5 nM WR99210 for six days and followed by addition of only 2.5 nM WR99210, generating Dd2^*Dd2*^and Dd2^*Dd2 L272F*^ parasites, respectively. Clones were established from the bulk cultures by limiting dilution^52^. PCR primers for verification of parental, recombinant control, and Dd2^*Dd2 L272F*^ parasite clones are shown in Supplementary Table S2.

### Expression of the C101F and L272F variants of PfCRT in *X. laevis* oocytes and measurements of CQ transport

Ethical approval of the work performed with the *X. laevis* frogs was obtained from the Australian National University Animal Experimentation Ethics Committee (Animal Ethics Protocol Number A2013/13) in accordance with the Australian Code of Practice for the Care and Use of Animals for Scientific Purposes. The C101F PfCRT^Dd2^, L272F PfCRT^Dd2^, and L272F PfCRT^3D7^ coding sequences were generated via site-directed mutagenesis using an approach described previously^32^. The mutations were introduced into codon-harmonized versions of the PfCRT^Dd2^ and PfCRT^3D7^ coding sequences, which encode retention motif-free forms of these proteins that are expressed at the plasma membrane of *X. laevis* oocytes^7^. All of the resulting coding sequences were verified by sequencing. The *in vitro* transcription of cRNA and the harvest and preparation of oocytes were performed as outlined elsewhere^67^. The oocytes were microinjected with 20 ng of cRNA and the uptake of [^3^H]CQ (0.25 μM; 20 Ci/mmol; American Radiolabeled Chemicals) was measured 3**–**4 days post-injection as detailed previously^67^. The measurements were made over 1.5 h at 27.5 °C and in medium that, unless otherwise specified, contained 96 mM NaCl, 2 mM KCl, 2 mM MgCl_2_, 1.8 mM CaCl_2_, 10 mM MES, 10 mM Tris**·**base (pH 5.5), and 15 μM unlabeled CQ. In all cases, at least three separate experiments were performed (on oocytes from different frogs), and in each experiment measurements were made from 10 oocytes per treatment.

### Immunofluorescence and western blot analyses of oocytes expressing PfCRT

Immunofluorescence analyses were performed on oocytes 3 days post-injection using an approach described elsewhere^68^. Briefly, the oocytes were fixed and labeled with rabbit anti-PfCRT antibody (concentration of 1:100; Genscript^32^; ) and Alexa Fluor 488 goat anti-rabbit antibody (concentration of 1:500; Molecular Probes). The oocytes were embedded in an acrylic resin using the Technovit 7100 plastic embedding system (Kulzer) as outlined previously^69^ and images of 4 μm slices were obtained with a Leica Microsystems inverted confocal laser microscope. At least two separate experiments were performed (on oocytes from different frogs) for each treatment and slices were examined from a minimum of three oocytes within a treatment. All of the slices taken from oocytes expressing a PfCRT variant displayed a fluorescent band above the pigment layer (consistent with the localization of PfCRT to the plasma membrane) that was not present in non-injected oocytes.

The preparation of oocyte membranes and the semi-quantification of PfCRT protein was carried out using a protocol described in detail elsewhere^32^. Protein samples prepared from oocyte membranes were separated on a 4–14% bis-Tris SDS-polyacrylamide gel (Life Technologies) and transferred to nitrocellulose membranes. The membranes were probed with rabbit anti-PfCRT antibody (1:4,000) followed by horseradish peroxidase-conjugated goat anti-rabbit antibody (1:8,000; Life Technologies). The PfCRT band for each variant was detected by chemiluminescence (Pierce), quantified using the Image J software^70^, and expressed as a percentage of the intensity measured for the PfCRT^Dd2^ band. In all cases, at least three separate experiments were performed (on oocytes from different frogs), and in each experiment measurements were averaged from two independent replicates.

### Curve fitting and statistical analyses

Mean half-maximal inhibitory concentrations (IC_50_ values) were derived by plotting percent growth inhibition against log drug concentration, and fitting the response data to a variable slope, sigmoidal curve-fit function for normalized data using Prism 5.0d for Macintosh (GraphPad Software). IC_50_ values represent means ± standard error from 3 independent tests. IC_50_ values between mutant and parent lines were tested for statistically significant differences using an *F*-test that determines whether the two dose response data sets are best described by single or independent curve fits (*p* < 0.05). In the case of the oocyte data, statistical comparisons were made using ANOVA in conjunction with Tukey’s multiple comparisons test. Other data were compared using the Student’s *t*-test and Fisher’s exact test as noted.

## Additional Information

**How to cite this article**: Pulcini, S. *et al.* Mutations in the *Plasmodium falciparum* chloroquine resistance transporter, PfCRT, enlarge the parasite's food vacuole and alter drug sensitivities. *Sci. Rep.* **5**, 14552; doi: 10.1038/srep14552 (2015).

## Supplementary Material

Supplementary Information

## Figures and Tables

**Table 1:** *In vitro* sensitivity of 3D7, 3D7^*L272F*^, FCB and FCB^*C101F*^ (in presence or absence of verapamil, VP) to antimalarial drugs.

Drug†	Mean ± SEM IC_50_ values for individual parasite strains/lines‡					
	3D7	3D7^*L272F*^	FCB	FCB^*C101F*^	FCB + VP	FCB^*C101F*^ + VP
CQ	15 ± 1.8	6.1 ± 1.5*	187 ± 7.1	34 ± 1.8*	47 ± 1.2	14 ± 1.0
QN	176 ± 17	108 ± 13*	333 ± 5.8	220 ± 23*	161 ± 33	223 ± 31
QD	—	—	167 ± 13	62 ± 3.1*	46 ± 1.9	46 ± 2.9
MQ	87 ± 29	64 ± 17	13 ± 1.1	14 ± 0.9	8.8 ± 1.0	18 ± 2.0
AQ	25 ± 2.3	27 ± 2.1	—	—	—	—
MDAQ	14 ± 1.5	15 ± 1.5	52 ± 4.1	18 ± 1.0*	16 ± 1.0	9.1 ± 0.7
PPQ	18 ± 4.1	22 ± 5.3	12 ± 0.6	27 ± 1.8*	13 ± 0.4	25 ± 3.1
ART	3.5 ± 1.2	4.1 ± 1.6	13 ± 0.9	9.1 ± 0.7**	9.9 ± 0.9	9.1 ± 0.5
BSD (μM)	1.5 ± 0.6	47 ± 6.0*	—	—	—	—
AMT (μM)	—	—	5.6 ± 0.6	465 ± 54*	11 ± 0.5	687 ± 32

^†^CQ, chloroquine; QN, quinine; QD, quinidine; MQ, mefloquine; AQ, amodiaquine; MDAQ, monodesethyl amodiaquine; PPQ, piperaquine; ART, artemisinin; BSD, blasticidin; AMT, amantadine; VP, verapamil (used at 0.8 μM).

^‡^IC_50_ values are listed in nM, except where indicated, and are show as the mean ± SEM. n = 3 independent assays (each performed as a single replicate for FCB parasites and in quintuplicate for 3D7 parasites). Significantly different mean IC_50_ values relative to controls (*F*-test; *p < 0.05, **p < 0.01).

**Table 2:** *In vitro* sensitivity of Dd2, Dd2^*Dd2*^, Dd2^*Dd2 L272F*^ and GC03 to antimalarial drugs.

Drug†	Mean ± SEM IC_50_ values for individual parasite strains/lines‡			
	**Dd2**	**Dd2^*Dd2*^**	**Dd2^*Dd2 L272F*^**	**GC03**
CQ	97 ± 6.8	88 ± 6.8	20 ± 1.8*	13 ± 2.3
MDCQ	497 ± 43	440 ± 33	121 ± 9.0*	26 ± 3.0
MDAQ	45 ± 5.8	35 ± 3.6	22 ± 4.0**	18 ± 1.1
PPQ	32 ± 3.1	32 ± 5.8	39 ± 1.5	23 ± 5.3
ART	18 ± 1.1	18 ± 4.6	13 ± 1.9	15 ± 4.7
BSD	456 ± 46	631 ± 35***	708 ± 54	456 ± 59

^†^CQ, chloroquine; MDCQ, monodesethyl chloroquine; MDAQ, monodesethyl amodiaquine; PPQ, piperaquine; ART, artemisinin; BSD, blasticidin.

^‡^IC_50_ values are listed in nM and are shown as the mean ± SEM. n = 3 independent assays (each performed in duplicate). Significantly different mean IC_50_ values between Dd2^*Dd2*^ and Dd2^*Dd2 L272F*^ (*F*-test; *p < 0.0001, **p = 0.07) and between Dd2 and Dd2^*Dd2*^ (*F*-test; ***p = 0.038).
