# Supplementary material for: Mutations in the Plasmodium falciparum chloroquine resistance transporter, PfCRT, enlarge the parasite’s food vacuole and alter drug sensitivities
Source: Sci Rep. 2015 Sep 30;5:14552. doi: 10.1038/srep14552 (PMC4588581; doi:10.1038/srep14552)
Supplement: Supplementary Information [file srep14552-s1.pdf]

## Supplementary data:

# Mutations in the *Plasmodium falciparum* chloroquine resistance transporter, PfCRT, enlarge the parasite's food vacuole and alter drug sensitivities

**Serena Pulcini[1+], Henry M. Staines[1+], Andrew H. Lee[2], Sarah H. Shafik[3], Guillaume Bouyer[1,4,5], Catherine M. Moore[1], Daniel A. Daley[6], Matthew J. Hoke[6], Lindsey M. Altenhofen[7], Heather J. Painter[7], Jianbing Mu[8], David J. P. Ferguson[9], Manuel Llinás[7], Rowena E. Martin[3], David A. Fidock[2,10], Roland A. Cooper[6,11] & Sanjeev Krishna[1]***

[1] Institute for Infection and Immunity, St. George's, University of London, London SW17 0RE, UK.
[2] Department of Microbiology and Immunology, Columbia University Medical Center, New York, NY 10032, USA.
[3] Research School of Biology, Australian National University, Canberra, ACT 2601, Australia.
[4] Sorbonne Universités, UPMC Univ. Paris 06, UMR 8227, Integrative Biology of Marine Models, Comparative Physiology of Erythrocytes, Station Biologique de Roscoff, Roscoff, France.
[5] CNRS, UMR 8227, Integrative Biology of Marine Models, Comparative Physiology of Erythrocytes, Station Biologique de Roscoff, Roscoff, France.
[6] Department of Biological Sciences, Old Dominion University, Norfolk, VA 23529, USA.
[7] Department of Biochemistry and Molecular Biology and Center for Malaria Research, Pennsylvania State University, State College, Pennsylvania 16802, USA.
[8] Laboratory of Malaria and Vector Research, National Institute of Allergy and Infectious Diseases, National Institutes of Health, Rockville MD 20852, USA.
[9] Nuffield Department of Clinical Laboratory Sciences, University of Oxford, John Radcliffe Hospital, Oxford OX3 9DU, UK.
[10] Division of Infectious Diseases, Department of Medicine, Columbia University Medical Center, New York, NY 10032, USA.
[11] Department of Natural Sciences and Mathematics, Dominican University of California, San Rafael, CA 94901, USA.

**[+]these authors contributed equally to this work**

***corresponding. s.krishna@sgul.ac.uk**

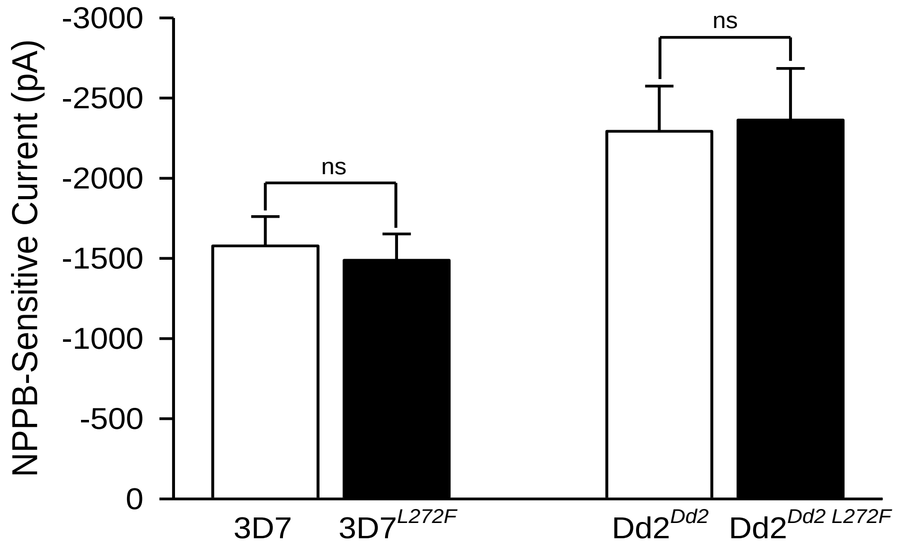


**Supplementary Figure S1 | Electrophysiological properties of parasite lines.** *Plasmodium* parasites are known to alter the permeability of their host's RBC plasma membrane[1]. This altered permeability is characterised by increased transport of a range of structurally unrelated solutes, with a preference for anions over electroneutral and cationic solutes[2]. Using electrophysiological technologies, infected RBCs exhibit greatly increased anionic currents, particularly at negative membrane potentials, that can be inhibited by non-specific anion transport blockers such as 5-nitro-2-(3-phenylpropylamino) benzoic acid (NPPB)[3]**.** BSD pressure has been shown to reduce parasite-induced RBC plasma membrane currents by altering expression of *clag3.1*[4, 5, 6], thus electrophysiological studies were undertaken to compare 3D7 and 3D7$^{L272F}$ and Dd2$^{Dd2}$ and Dd2$^{Dd2\ L272F}$-infected RBCs. The ruptured patch whole-cell voltage-clamp configuration was used to record membrane currents[7]. All experiments were performed at room temperature. Patch pipettes (tip resistances 6 to 12 MΩ) were prepared from borosilicate glass capillaries pulled and polished on a Werner Zeitz DMZ programmable puller. The bath solution contained 155 mM NaCl, 1.4 mM $CaCl_2$, 1 mM $MgCl_2$, 10 mM HEPES, 10 mM glucose (pH 7.4, 310 ± 5 mOsm/kg $H_2O$). The pipette solution was of the same composition, with the one exception that 1.4 mM $CaCl_2$ was replaced with 0.5 mM EGTA. A 2 ml aliquot of infected RBC suspension (0.001% hematocrit) was placed in a 35 mm Petri dish and left while the RBCs settled. Having obtained a seal (5-15 GΩ) on an infected RBC, the cell was lifted off the bottom of the dish and the patch ruptured by brief application of negative pressure, to attain the whole-cell configuration. Whole-cell currents were recorded using an Axopatch 200B amplifier (digitised at 10 kHz and filtered at 5 kHz with a 4-pole Bessel filter), with voltage command protocols generated and the currents analysed using the pCLAMP software suite (Version 10, Molecular Devices). Whole-cell current/voltage (I-V) curves were obtained by evoking a series of $V_T$ values from -100 to +100 mV in 10 mV steps for 300 ms from a $V_H$ of 0 mV. Current data were measured over the last 50 ms of each record (*i.e.* 250-300 ms). NPPB-sensitive currents at a $V_T$ of -100 mV are shown. NPPB was used at a concentration of 100 µM, which on average inhibited 94 ± 2% of the total current (n = 25). Current data were not included if NPPB inhibition was not greater than 75%, suggesting loss of seal. No significant differences (ns) were found between 3D7 (n = 5) and 3D7$^{L272F}$ (n = 5) and Dd2$^{Dd2}$ (n = 7) and Dd2$^{Dd2\ L272F}$-infected RBCs (n = 8), respectively, suggesting no alteration in the permeability of infected RBCs (Students's *t*-test; $p > 0.5$).

**a**

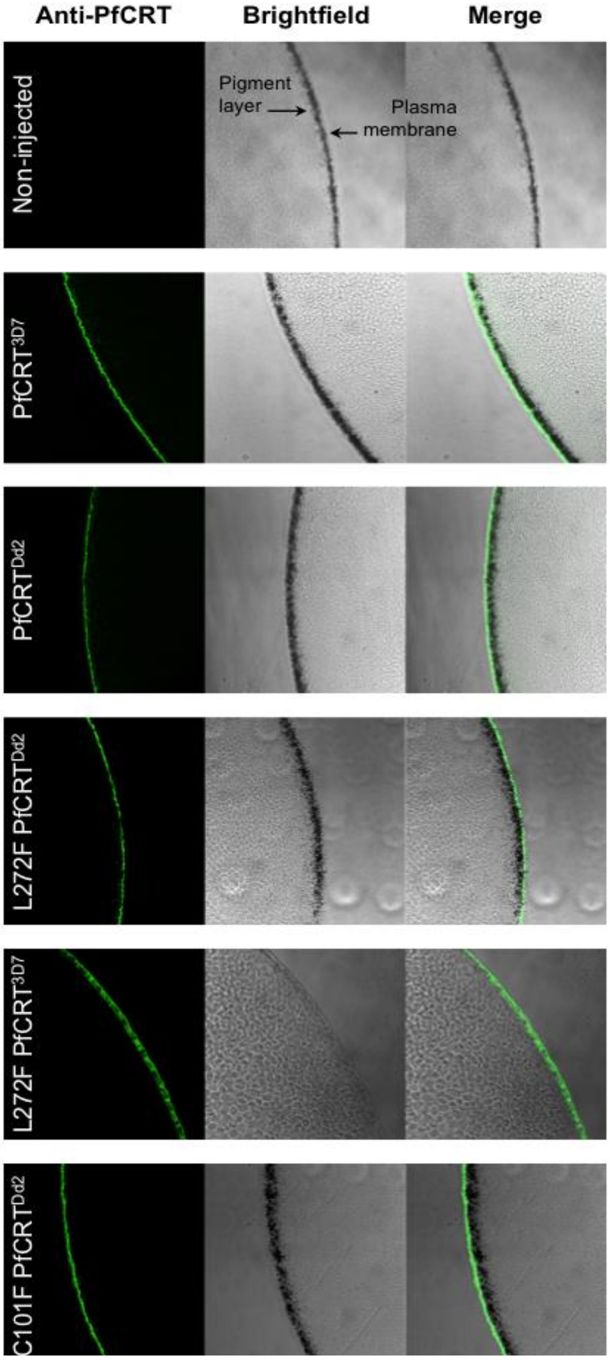


**b**

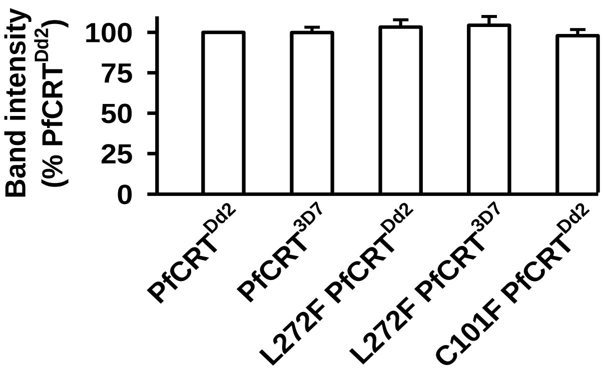


**Supplementary Figure S2 | Expression of the C101F and L272F variants of PfCRT in *Xenopus* oocytes.** (**a**) Immunofluorescence microscopy was used to localize L272F $PfCRT^{Dd2}$, L272F $PfCRT^{3D7}$, and C101F $PfCRT^{Dd2}$ in the oocyte. In each case, the expression of the PfCRT variant resulted in a fluorescent band external to the pigment layer, indicating that the protein was expressed in the oocyte plasma membrane. The band was not present in non-injected oocytes. (**b**) The level of PfCRT protein in the oocyte membrane was semiquantified using a western blot method[8]. The analysis included $PfCRT^{Dd2}$ as a control, to which the other band intensity values were normalized. Levels of PfCRT protein are shown as mean + SEM from three to five separate experiments (in each experiment measurements were averaged from two independent replicates). There were no significant differences in expression levels between constructs ($p > 0.05$); hence, all of the PfCRT variants were present at similar levels in the oocyte membrane.

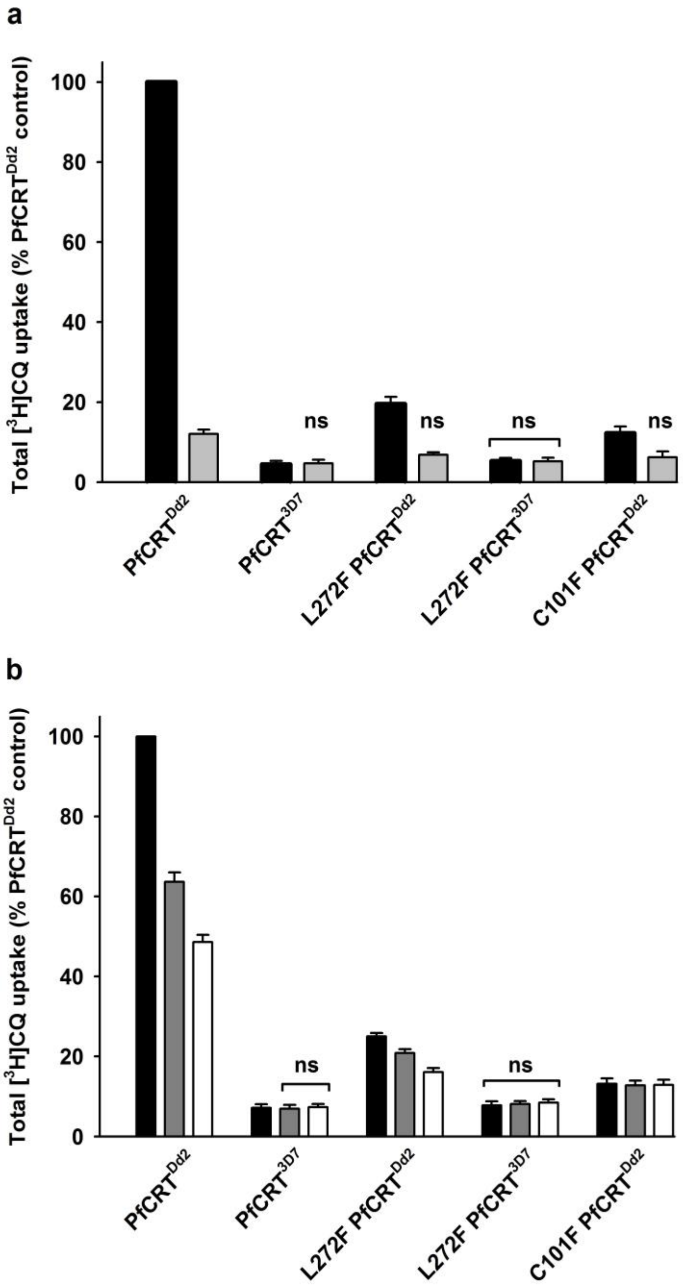


**Supplementary Figure S3 | Total CQ transport activity of the C101F and L272F variants of PfCRT in *Xenopus* oocytes.** (**a**,**b**) The uptake of [$^3$H]CQ into oocytes expressing PfCRT was measured in the absence (closed bars) or presence of 250 µM VP (light grey bars; **a**), 100 µM BSD (dark grey bars; **b**), or 500 µM BSD (open bars; **b**). Within each experiment, measurements were made from 10 oocytes per treatment and uptake was expressed relative to that measured in the PfCRT$^{Dd2}$-expressing oocytes under control conditions. The normalized data obtained from 4-5 separate experiments (each using oocytes from different frogs) were then averaged and are shown + SEM. These data are total CQ uptake (*i.e.* both CQ accumulation attributable to diffusion and that via PfCRT). In the control treatments, the rates of CQ uptake (pmol/oocyte/h; n = 9 ± SEM) in oocytes expressing PfCRT$^{Dd2}$ and PfCRT$^{3D7}$ were 23.6 ± 2.3 and 1.3 ± 0.2, respectively. 'ns' denotes no significant difference ($p > 0.05$) in CQ accumulation between oocytes expressing a PfCRT variant (in the presence or absence of VP or BSD) and that measured in the PfCRT$^{3D7}$-expressing oocytes under control conditions.

**Supplementary Table S1 | Primers used for sequencing of PfCRT in 3D7 parasites**

| Name | Sequence (5' to 3') | Direction | Product (bp) |
|---|---|---|---|
| **Crt1** | CCGTTAATAATAAATACACGCAG | Forward | 540 |
| **Crt2** | GTTCTTGTAAGACCTATGAAGGCC | Reverse | |
| **Crt3** | ATCCATGTTAGATGCCTGTTCAGTC | Forward | 479 |
| **Crt4** | CCCAAGAATAAACATGCGAAACC | Reverse | |
| **Crt5** | GCTTTTCAAACATGACAAGGG | Forward | 581 |
| **Crt6** | CGACGTTGGTTAATTCTCCTTC | Reverse | |
| **Crt7** | GTCTTATATTACCTGTATACACCC | Forward | 600 |
| **Crt8** | CCTTATAAAGTGTAATGCGATAGC | Reverse | |

**Supplementary Table S2 | PCR verification primers for transfection experiments**

| Name | Sequence (5' to 3') |
|---|---|
| **p3264** | CTTGAATTCGACCTTAACAGATGGCTCAC |
| **p3265** | CTTATCGATAAGCAGAAGAACATATTAATAGGAATACTTAATTG |
| **p3315** | CTCGAGATGGTTGGTTCGCTAAACTGC |
| **p3403** | TTGACCCTTATATATTCCACCCA |
| **p3404** | CTTGGGCCCAAGTTGTACTGCTTCTAAGC |
| **p3527** | CCTGTATACACCCTTCCATTTTTAAAAGAATTTCATTTACCATATAATGAAATATGGAC |
| **p3528** | GTCCATATTTCATTATATGGTAAATGAAATTCTTTTAAAAATGGAAGGGTGTATACAGG |

**References - Supplementary data**


1. Ginsburg, H., Krugliak, M., Eidelman, O. & Cabantchik, Z. I. New permeability pathways induced in membranes of *Plasmodium falciparum*-infected erythrocytes. *Mol Biochem Parasitol* **8**, 177-190 (1983).

2. Kirk, K., Horner, H. A., Elford, B. C., Ellory, J. C. & Newbold, C. I. Transport of diverse substrates into malaria-infected erythrocytes via a pathway showing functional characteristics of a chloride channel. *J Biol Chem* **269**, 3339-3347 (1994).

3. Staines, H. M. *et al.* Electrophysiological studies of malaria parasite-infected erythrocytes: current status. *Int J Parasitol* **37**, 475-482 (2007).

4. Hill, D. A. *et al.* A blasticidin S-resistant *Plasmodium falciparum* mutant with a defective plasmodial surface anion channel. *Proc Natl Acad Sci U S A* **104**, 1063-1068 (2007).

5. Mira-Martinez, S. *et al.* Epigenetic switches in *clag3* genes mediate blasticidin S resistance in malaria parasites. *Cell Microbiol* **15**, 1913-1923 (2013).

6. Sharma, P. *et al.* An epigenetic antimalarial resistance mechanism involving parasite genes linked to nutrient uptake. *J Biol Chem* **288**, 19429-19440 (2013).

7. Staines, H. M. *et al.* Solute transport via the new permeability pathways in *Plasmodium falciparum*-infected human red blood cells is not consistent with a simple single-channel model. *Blood* **108**, 3187-3194 (2006).

8. Summers, R. L. *et al.* Diverse mutational pathways converge on saturable chloroquine transport via the malaria parasite's chloroquine resistance transporter. *Proc Natl Acad Sci U S A* **111**, E1759-1767 (2014).
